# PARTNERING WITH NURSING HOME PROVIDERS FOR PERSON-CENTERED QUALITY IMPROVEMENT

**DOI:** 10.1093/geroni/igz038.194

**Published:** 2019-11-08

**Authors:** Abigail Hermesch, Da Jung Chang, Chelsea Goldstein, Kimberly Van Haitsma, Katherine Abbott

**Affiliations:** 1 Miami University, Oxford, Ohio, United States; 2 Scripps Gerontology Center at Miami University, Oxford, Ohio, United States; 3 The Pennsylvania State University, University Park, Pennsylvania, United States

## Abstract

The purpose of this study was to assist Ohio NH providers in implementing PAL Cards. Providers were recruited to utilize the recreation and leisure items from the PELI to assess resident important preferences and create PAL cards for 15-20 residents. A total of n=43 providers registered and n=26 (60%) providers completed the project, which involved monthly coaching calls and an end of project telephone interview. Participating providers were not for profit 46% (n=12), for-profit 46% (n=12), and government-owned 8% (n=2). Their average bed size was 87 and 50% (n=13) of providers had five-star ratings with a range from 1-5. Participants attempted n=439 PAL card interviews with residents and completed n=414 (94.31%) PAL Cards. Providers received over 1,428 minutes of coaching support from the Project Manager over the phone. The majority of providers were successful in implementing PAL Cards for residents with substantial support from the project manager.

